# gutSMASH 2.0: Extended Identification of Primary Metabolic Gene Clusters From the Human Gut Microbiota ^☆^

**DOI:** 10.1016/j.jmb.2025.169567

**Published:** 2025-11-28

**Authors:** Yijun Zhu, Hannah E. Augustijn, Victòria Pascal Andreu, Arjan Draisma, Gilles P. van Wezel, Dylan Dodd, Michael A. Fischbach, Marnix H. Medema

**Affiliations:** 1 - **Institute of Precision Medicine,** The First Affiliated Hospital, Sun Yat-sen University, Guangzhou 510080, China; 2 - **Bioinformatics Group,** Wageningen University, Wageningen, The Netherlands; 3 - **Institute of Biology**, Leiden University, Leiden, The Netherlands; 4 - **Institut Germans Trias i Pujol,** Epigenetic Mechanisms of Cancer and Cell Differentiation Group, Barcelona, Spain; 5 - **Department of Pathology,** Stanford University, Stanford, USA; 6 - **Department of Microbiology & Immunology,** Stanford University, Stanford, USA; 7 - **Department of Bioengineering,** Stanford University, Stanford, USA; 8 - **Chan Zuckerberg Biohub,** San Francisco, CA, USA

**Keywords:** metabolic gene cluster, gut microbiome, genome analysis, web server

## Abstract

Microbiota-derived metabolites serve as key messengers mediating host–microbe and microbe–microbe interactions, often through specialized primary metabolic pathways. gutSMASH was initially developed to systematically identify the metabolic gene clusters (MGCs) that encode these pathways in anaerobic gut microbial genomes. Here, we present gutSMASH 2.0, a major update that significantly expands its functionality. This version introduces 14 new detection rules covering 12 additional types of MGCs. The comparative genomics framework was enhanced with 26 experimentally validated MGCs and 15,024 gene clusters from the Cultivated Genome Reference 2 (CGR2) collection. Furthermore, gutSMASH 2.0 integrates transcription factor binding site prediction using LogoMotif’s methodology, enabling investigation of MGC regulatory elements. Together, these improvements make gutSMASH a more powerful tool for automated discovery and analysis of niche-determining metabolic pathways in the gut microbiome. gutSMASH 2.0 is freely available at https://gutsmash.bioinformatics.nl/.

## Introduction

Microorganisms synthesize a wide array of small molecules through their primary and secondary metabolic pathways. These metabolites play essential roles in mediating ecological interactions with other microbes and in shaping host–microbe relationships [1,2]. Over the past decade, genome mining has become an essential approach for exploring microbial metabolic diversity, enabling the discovery of novel bioactive compounds and their biosynthetic gene clusters (BGCs) [3,4].

gutSMASH was developed as an automated tool for the identification and analysis of metabolic gene clusters (MGCs) involved in niche-determining specialized primary metabolism in gut microbiota [5,6]. Since the release of the original gutSMASH in 2023, the landscape of microbial-derived metabolites has expanded significantly, with increasing numbers of unique compounds and their corresponding biosynthetic genes being characterized. A critical next step in understanding microbial metabolism is elucidating how MGCs are transcriptionally regulated. Reliable prediction of MGC regulation requires deeper insight into transcription factor binding sites (TFBSs) and the regulatory hierarchies that control both global and specific gene expression patterns [7].

To address these needs, we present gutSMASH version 2.0, released in December 2024. This updated version introduces 14 new detection rules covering 12 additional MGC types, including those involved in the biosynthesis, breakdown, or modification of health-related metabolites such as H_2_S [8], uric acid [9,10], TMAO [11,12], phenylacetylglutamine [13], and bilirubin [14]. The comparative genomic database has been significantly expanded to include 26 experimentally validated MGCs and 15,024 predicted gene clusters from the Cultivated Genome Reference 2 (CGR2) collection. Furthermore, TF binding site predictions, powered by LogoMotif and MiniMotif [7], have been integrated into the pipeline, enabling the analysis of MGC regulatory mechanisms. An overview of the major updates is provided in Table 1, and detailed release notes are available at https://gutsmash.bioinformatics.nl.

## Main Updates

### New features and updates

Since gutSMASH 1.0 was released, recent studies have revealed additional gut microbiome–mediated metabolic pathways associated with host health and disease, with mechanistic insights emerging for sulfur, uric acid, bilirubin, amino acid, and carnitine metabolism [8,9,11,12,14]. While gutSMASH 1.0 briefly addressed these processes, version 2.0 provides a comprehensive catalog of sulfur metabolism and sulfide-generating gene clusters, including pathways for sulfate, taurine, and sulfoquinovose utilization [8,15]. Beyond sulfur metabolism, gutSMASH 2.0 introduces rules for uric acid metabolism, underscoring the critical role of the gut microbiome in compensating for the human lack of uricase [9,10]. Furthermore, we incorporated detection of additional physiologically relevant metabolic gene clusters, including phenylpyruvate:-ferredoxin oxidoreductase, bilirubin reductase [14], and r-butyrobetaine [11,12] utilization, which generate phenylacetic acid [13], urobilinogen, and trimethylamine, respectively.

**Figure 1.**
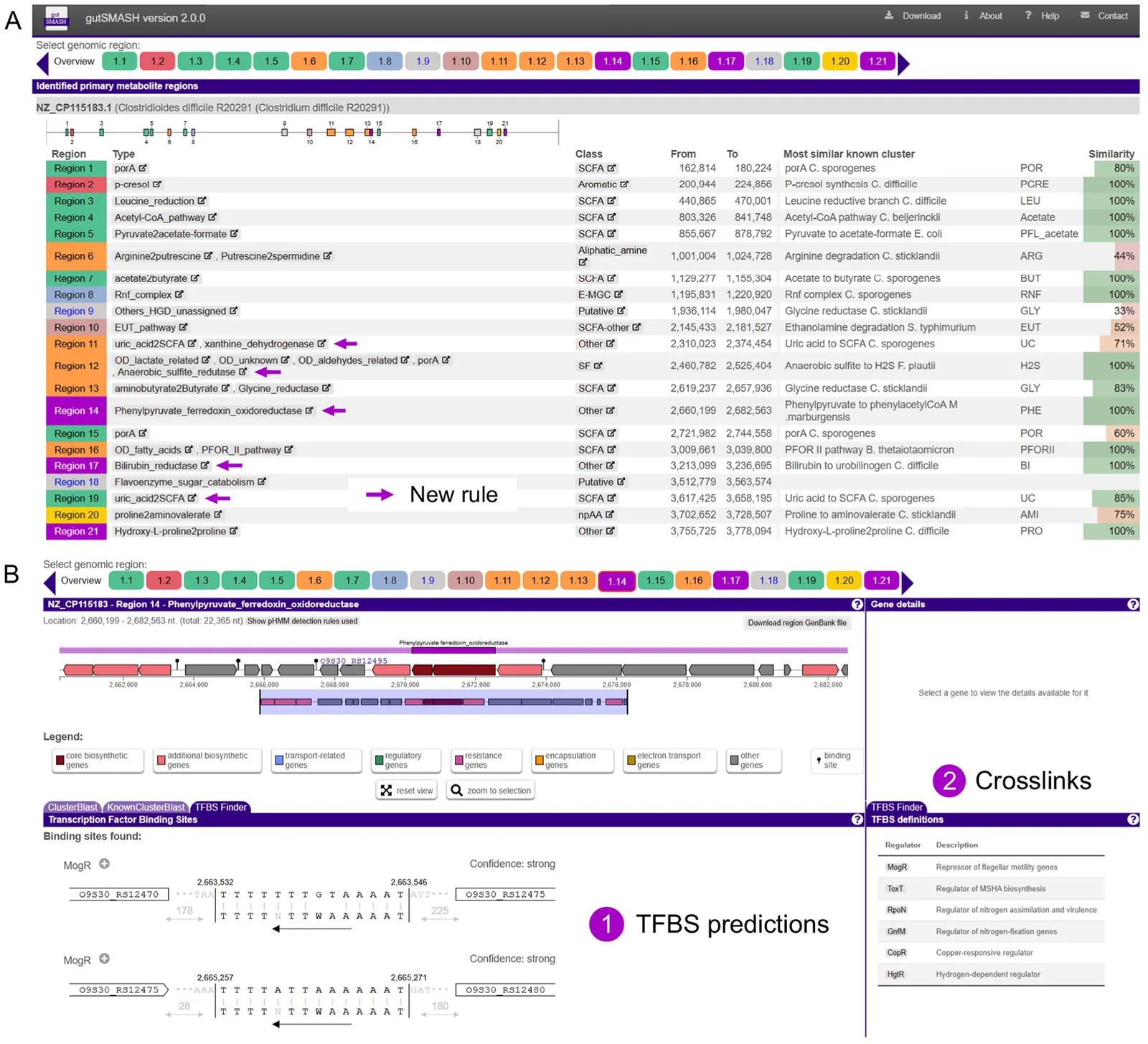
Gutsmash 2.0 run for *Clostridioides difficile* R20291 (NZ_CP115183.1). (A) Predicted MGCs. MGCs detected by the new rules are indicated with arrows. (B) Cluster visualization with integrated transcription factor binding site (TFBS) predictions. 1, Detected TFBS matches to the prediction matrix; 2, A short description of each detected TF with crosslinks to their source database for more information.

In gutSMASH version 2.0, we expanded the coverage by incorporating additional experimentally characterized bacterial metabolic gene clusters (MGCs) from 51 to 63 and enhancing detection rules from 41 to 55, thereby improving the profiling of microbial metabolic potential (Table 1, Figure 1A). Moreover, gutSMASH 2.0 integrates transcription factor binding site (TFBS) predictions through LogoMotif and MiniMotif [7], offering users valuable insights into the regulatory mechanisms of target genes and enhancing genome mining strategies (Table 1, Figure 1B). Given that the core algorithm for MGC detection and annotation is consistent across both versions, their computational runtimes are comparable.

### Transcription factor binding site prediction for insights into target gene regulation

In gutSMASH 2.0, we now offer the option to detect TF binding sites through the TFBS Finder module (Figure 1B). This module builds on the TFBS prediction algorithm first implemented in antiSMASH 7.0 [16] and relies on the MiniMotif prediction algorithm, part of the LogoMotif database [7]. As LogoMotif is primarily focused on Actinobacteria, the underlying dataset has been extended with TFs from a broader range of gram-positive and gram-negative bacteria, including *Pseudomonas* spp., *Escherichia coli*, and *Lactococcus* spp., derived from the CollecTF database [17]. Through this expansion, 50 matrices are available for TFBS detection, offering insights into the regulatory mechanisms governing gene expression in the given MGC.

### More comprehensive gene functional annotations help to get more insights on the reaction

**Figure 2.**
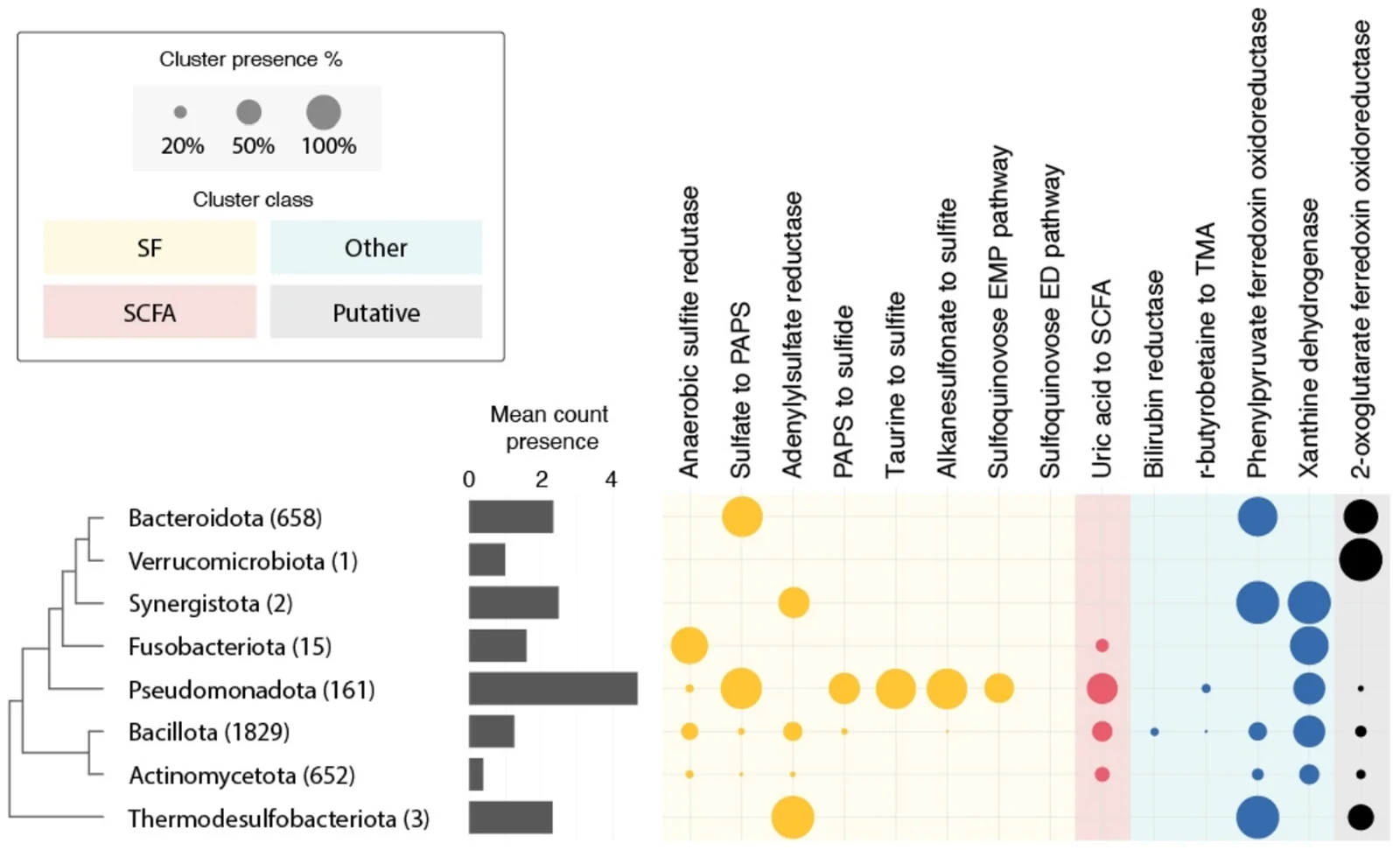
Distribution of newly added metabolic pathways across the most representative genera in the human gut CGR2 genome collections. Circles represent the absence/presence of known pathways in each genus. The colors indicate the four cluster classes: sulfur metabolism (SF), short chain fatty acids (SCFA), putative and other. The tree was generated using phyloT (https://phylot.biobyte.de/) and visualized using iTOL [19]. Raw data are available in Supplementary Table 2.

To profile the metabolic capacity of strains within the human gut microbiome, we selected 2604 high-quality reference genomes from the Culturable Genome Reference collection 2 (CGR2) [9,18]. Using gutSMASH 2.0, we predicted 15,024 metabolic gene clusters (MGCs) across the CGR2 genomes that are clear homologs of known metabolic pathways. Bacteria from different taxonomic lineages exhibited distinct metabolic capacities [5]. For example, anaerobic sulfite reductase was detected in over 80% of Fusobacteriota genomes, while taurine-to-sulfite metabolism mediated by the *tauABCD* operon was predominantly found in Pseudomonadota (>80%) (Figure 2).

### Conclusion and perspectives

Bacteria produce a vast array of natural products with applications in clinical, agricultural, and biotechnological contexts. As more physiologically relevant microbially derived metabolites are identified, evaluating microbial metabolic potential through genomic data becomes increasingly essential. The gutSMASH platform offers a comprehensive, automated tool for identifying niche-defining primary metabolic pathways from genome sequences or metagenomic contigs. Many genes involved in these pathways are tightly regulated by TFs and their associated TFBSs, enabling bacteria to dynamically respond to environmental changes. The integration of TFBS prediction into metabolic analysis provides a valuable framework for uncovering regulatory mechanisms and broadens the platform’s applicability across diverse areas of microbiome research. Expanding the platform to allow queries by taxonomic rank (e.g., species or genus) or retrieval of gene clusters based on specific protein family combinations would further enhance its functionality.

## Materials and Methods

gutSMASH is a Python-based pipeline that was built from antiSMASH version 5.0 source code. The latest command line version is freely available and can be downloaded and installed at https://github.com/victoriapascal/gutsmash/tree/gutsmash.

### Finding pathway signatures for known and characterized MGCs

To create a new set of detection rules, 26 known and characterized MGCs were gathered from the literature and used as positive controls. The CUTOFF and NEIGHBORHOOD parameters were optimized and subsequently validated against positive controls (Supplementary Table 1). The protein sequences of these MGCs were searched using hmmscan (HMMER suit version 3.4, 15 August 2023; https://hmmer.org/). From the resulting pHMM hits, auxiliary and core domains were manually identified for each pathway, to ultimately determine the pathway signature and specify it in the corresponding detection rule. To discern and more precisely identify key enzymes of interest sharing a keystone domain, we used custom-made pHMMs described in Andreu et al (2023) [5]. Altogether, the knowledge on the core enzyme-coding genes and the newly built pHMMs helped to construct a preliminary set of detection rules to predict known pathways.

### Transcription factor binding site predictions

The implementation of transcription factor binding site (TFBS) prediction within the framework builds upon the methodology introduced in LogoMotif [7], and the TFBS Finder module integrated in antiSMASH v7 [16]. Briefly, this module employs experimentally validated TFBSs to generate position weight matrices (PWMs), which are constructed using Shannon entropy calculations. While the TFBS Finder in antiSMASH v7 relied on the 17 PWMs available in the LogoMotif database, we expanded this collection by incorporating an additional 33 PWMs from the CollecTF database [17]. This extension provides broader coverage of regulatory motifs relevant to bacterial taxa within the gut microbiome. Each PWM was manually curated and quality-checked in accordance with the procedures described in the original LogoMotif publication. Detection of putative TFBSs in intergenic regions of predicted metabolic gene clusters (MGCs) is performed using MOODS [20], with a default threshold of *p* ≤ 1 × 10^−5^ and a maximum 50 bp overlap with adjacent coding sequences. Identified matches are reported both in the JSON output files and visually represented in the gutSMASH web interface. Specifically, results are displayed as pin markers within the detected MGCs and in dedicated side panels that provide detailed information, including TFBS alignment visualizations, PWM match scores, and interactive gene selection options. Crosslinks to the originating databases are also provided for further functional annotation and biological interpretations.

## Supplementary Material

1

2

## Figures and Tables

**Table 1:** New features of gutSMASH 2.0.

Items	gutSMASH 1.0	gutSMASH 2.0
KnownClusterBlast: known metabolic gene clusters	59	83
Distinct primary metabolic pathways covered	51	63
Detection rules	41	55
TFBS prediction matrices	NA	50

## Data Availability

Overview of pathways encoded by genes and MGCs, and key enzyme-coding genes with their associated Pfam domains are listed in Supplementary Table 1. Taxonomic assignments of bacteria were performed according to Genome Taxonomy Database release 95 (https://gtdb.ecogenomic.org/). Lists of accessions of genome assemblies used are available in Supplementary Table 2. The gutSMASH source code is available freely under an open-source AGPL-3.0 license from https://github.com/victoriapascal/gutsmash/.

## Appendix A. Supplementary material

Supplementary material to this article can be found online at https://doi.org/10.1016/j.jmb.2025.169567.

## References

[1] Iliev ID, Ananthakrishnan AN, Guo CJ, (2025). Microbiota in inflammatory bowel disease: mechanisms of disease and therapeutic opportunities. Nature Rev. Microbiol. 23, 509–524. 10.1038/s41579-025-01163-0. 40065181 PMC12289240

[2] Brown EM, Clardy J, Xavier RJ, (2023). Gut microbiome lipid metabolism and its impact on host physiology. Cell Host Microbe 31, 173–186. 10.1016/j.chom.2023.01.009. 36758518 PMC10124142

[3] Augustijn HE, Reitz ZL, Zhang L, Boot JA, Elsayed SS, Challis GL, Medema MH, van Wezel GP, (2025). Genome mining based on transcriptional regulatory networks uncovers a novel locus involved in desferrioxamine biosynthesis. PLoS Biol. 23, e3003183. 10.1371/journal.pbio.3003183. 40504771 PMC12161575

[4] Tran PN, Yen MR, Chiang CY, Lin HC, Chen PY, (2019). Detecting and prioritizing biosynthetic gene clusters for bioactive compounds in bacteria and fungi. Appl. Microbiol. Biotechnol. 103, 3277–3287. 10.1007/s00253-019-09708-z. 30859257 PMC6449301

[5] Pascal Andreu V, Augustijn HE, Chen L, Zhernakova A, Fu J, Fischbach MA, Dodd D, Medema MH, (2023). gutSMASH predicts specialized primary metabolic pathways from the human gut microbiota. Nature Biotechnol. 41, 1416–1423. 10.1038/s41587-023-01675-1. 36782070 PMC10423304

[6] Pascal Andreu V, Roel-Touris J, Dodd D, Fischbach MA, Medema MH, (2021). The gutSMASH web server: automated identification of primary metabolic gene clusters from the gut microbiota. Nucleic Acids Res. 49, W263–W270. 10.1093/nar/gkab353. 34019648 PMC8262752

[7] Augustijn HE, Karapliafis D, Joosten KMM, Rigali S, van Wezel GP, Medema MH, (2024). LogoMotif: a comprehensive database of transcription factor binding site profiles in Actinobacteria. J. Mol. Biol. 436, 168558. 10.1016/j.jmb.2024.168558. 38580076

[8] Luo W, Zhao M, Dwidar M, Gao Y, Xiang L, Wu X, Medema MH, Xu S, Li X, Schafer H, Chen M, Feng R, Zhu Y, (2024). Microbial assimilatory sulfate reduction-mediated H(2)S: an overlooked role in Crohn’s disease development. Microbiome 12, 152. 10.1186/s40168-024-01873-2. 39152482 PMC11328384

[9] Liu Y, Jarman JB, Low YS, Augustijn HE, Huang S, Chen H, DeFeo ME, Sekiba K, Hou BH, Meng X, Weakley AM, Cabrera AV, Zhou Z, van Wezel G, Medema MH, Ganesan C, Pao AC, Gombar S, Dodd D, (2023). A widely distributed gene cluster compensates for uricase loss in hominids. Cell 186, 3400–3413.e3420. 10.1016/j.cell.2023.06.010. 37541197 PMC10421625

[10] Liu Y, Zhou Z, Jarman JB, Chen H, Miranda-Velez M, Terkeltaub R, Dodd D, (2025). Gut bacteria degrade purines via the 2,8-dioxopurine pathway. Nature Microbiol. 10, 2291–2305. 10.1038/s41564-025-02079-4. 40770490 PMC12666987

[11] Buffa JA, Romano KA, Copeland MF, Cody DB, Zhu W, Galvez R, Fu X, Ward K, Ferrell M, Dai HJ, Skye S, Hu P, Li L, Parlov M, McMillan A, Wei X, Nemet I, Koeth RA, Li XS, Wang Z, Sangwan N, Hajjar AM, Dwidar M, Weeks TL, Bergeron N, Krauss RM, Tang WHW, Rey FE, DiDonato JA, Gogonea V, Gerberick GF, Garcia-Garcia JC, Hazen SL, (2022). The microbial gbu gene cluster links cardiovascular disease risk associated with red meat consumption to microbiota L-carnitine catabolism. Nature Microbiol. 7, 73–86. 10.1038/s41564-021-01010-x. 34949826 PMC8732312

[12] Dwidar M, Buffa JA, Wang Z, Santos A, Tittle AN, Fu X, Hajjar AM, DiDonato JA, Hazen SL, (2023). Assembling the anaerobic gamma-butyrobetaine to TMA metabolic pathway in *Escherichia fergusonii* and confirming its role in TMA production from dietary L-carnitine in murine models. MBio 14, e0093723. 10.1128/mbio.00937-23. 37737636 PMC10653785

[13] Zhu Y, Dwidar M, Nemet I, Buffa JA, Sangwan N, Li XS, Anderson JT, Romano KA, Fu X, Funabashi M, Wang Z, Keranahalli P, Battle S, Tittle AN, Hajjar AM, Gogonea V, Fischbach MA, DiDonato JA, Hazen SL, (2023). Two distinct gut microbial pathways contribute to meta-organismal production of phenylacetylglutamine with links to cardiovascular disease. Cell Host Microbe 31, 18–32. e19. 10.1016/j.chom.2022.11.015. 36549300 PMC9839529

[14] Hall B, Levy S, Dufault-Thompson K, Arp G, Zhong A, Ndjite GM, Weiss A, Braccia D, Jenkins C, Grant MR, Abeysinghe S, Yang Y, Jermain MD, Wu CH, Ma B, Jiang X, (2024). BilR is a gut microbial enzyme that reduces bilirubin to urobilinogen. Nature Microbiol. 9, 173–184. 10.1038/s41564-023-01549-x. 38172624 PMC10769871

[15] Krasenbrink J, Hanson BT, Weiss AS, Borusak S, Tanabe TS, Lang M, Aichinger G, Hausmann B, Berry D, Richter A, Marko D, Mussmann M, Schleheck D, Stecher B, Loy A, (2025). Sulfoquinovose is exclusively metabolized by the gut microbiota and degraded differently in mice and humans. Microbiome 13, 184. 10.1186/s40168-025-02175-x. 40775374 PMC12330013

[16] Blin K, Shaw S, Augustijn HE, Reitz ZL, Biermann F, Alanjary M, Fetter A, Terlouw BR, Metcalf WW, Helfrich EJN, van Wezel GP, Medema MH, Weber T, (2023). antiSMASH 7.0: new and improved predictions for detection, regulation, chemical structures and visualisation. Nucleic Acids Res. 51, W46–W50. 10.1093/nar/gkad344. 37140036 PMC10320115

[17] Kilic S, White ER, Sagitova DM, Cornish JP, Erill I, (2014). CollecTF: a database of experimentally validated transcription factor-binding sites in Bacteria. Nucleic Acids Res. 42, D156–D160. 10.1093/nar/gkt1123. 24234444 PMC3965012

[18] Zou Y, Xue W, Luo G, Deng Z, Qin P, Guo R, Sun H, Xia Y, Liang S, Dai Y, Wan D, Jiang R, Su L, Feng Q, Jie Z, Guo T, Xia Z, Liu C, Yu J, Lin Y, Tang S, Huo G, Xu X, Hou Y, Liu X, Wang J, Yang H, Kristiansen K, Li J, Jia H, Xiao L, (2019). 1,520 reference genomes from cultivated human gut bacteria enable functional microbiome analyses. Nature Biotechnol. 37, 179–185. 10.1038/s41587-018-0008-8. 30718868 PMC6784896

[19] Letunic I, Bork P, (2021). Interactive Tree Of Life (iTOL) v5: an online tool for phylogenetic tree display and annotation. Nucleic Acids Res. 49, W293–W296. 10.1093/nar/gkab301. 33885785 PMC8265157

[20] Korhonen J, Martinmaki P, Pizzi C, Rastas P, Ukkonen E, (2009). MOODS: fast search for position weight matrix matches in DNA sequences. Bioinformatics 25, 3181–3182. 10.1093/bioinformatics/btp554. 19773334 PMC2778336

